# Advanced Glycation End Products Are Retained in Decellularized Muscle Matrix Derived from Aged Skeletal Muscle

**DOI:** 10.3390/ijms22168832

**Published:** 2021-08-17

**Authors:** Lucas C. Olson, Tri M. Nguyen, Rebecca L. Heise, Barbara D. Boyan, Zvi Schwartz, Michael J. McClure

**Affiliations:** 1 Department of Biomedical Engineering, College of Engineering, Virginia Commonwealth University, Richmond, VA 23284, USA; olsonlc@vcu.edu (L.C.O.); tmnguyen2@vcu.edu (T.M.N.); rlheise@vcu.edu (R.L.H.); bboyan@vcu.edu (B.D.B.); zschwartz@vcu.edu (Z.S.); 2 Wallace H. Coulter Department of Biomedical Engineering, Georgia Institute of Technology, Atlanta, GA 30332, USA; 3 Department of Periodontics, University of Texas Health Science Center at San Antonio, San Antonio, TX 78229, USA

**Keywords:** skeletal muscle aging, decellularization, advanced glycation end products, collagen cross-linking

## Abstract

Decellularized tissues are biocompatible materials that engraft well, but the age of their source has not been explored for clinical translation. Advanced glycation end products (AGEs) are chemical cross-links that accrue on skeletal muscle collagen in old age, stiffening the matrix and increasing inflammation. Whether decellularized biomaterials derived from aged muscle would suffer from increased AGE collagen cross-links is unknown. We characterized gastrocnemii of 1-, 2-, and 20-month-old *C57BL/6J* mice before and after decellularization to determine age-dependent changes to collagen stiffness and AGE cross-linking. Total and soluble collagen was measured to assess if age-dependent increases in collagen and cross-linking persisted in decellularized muscle matrix (DMM). Stiffness of aged DMM was determined using atomic force microscopy. AGE levels and the effect of an AGE cross-link breaker, ALT-711, were tested in DMM samples. Our results show that age-dependent increases in collagen amount, cross-linking, and general stiffness were observed in DMM. Notably, we measured increased AGE-specific cross-links within old muscle, and observed that old DMM retained AGE cross-links using ALT-711 to reduce AGE levels. In conclusion, deleterious age-dependent modifications to collagen are present in DMM from old muscle, implying that age matters when sourcing skeletal muscle extracellular matrix as a biomaterial.

## 1. Introduction

Musculoskeletal aging in individuals over 65 years impacts independence, increases the risk of fall-related injuries, and reduces life quality [1]. The deterioration of muscle’s strength and mass with increasing age culminates in a condition known as sarcopenia, an age-dependent muscle wasting disease [2,3,4,5,6,7]. Advanced glycation end products (AGEs), the final derivative of the Maillard or browning reaction, are known to accumulate in musculoskeletal tissues in old age and are thought to play a role in the development of sarcopenia [8,9,10]. AGEs preferentially accrue on the long-lived extracellular matrix (ECM) proteins, especially collagens, since their formation relies on its precursors’ stochastic reaction (glucose and proteins) via the Maillard reaction. In addition to having a long half-life, collagens are rich in repeating arginine and lysine amino acids that potentiate the reaction between collagen and AGE precursors, further predisposing collagen to these glycation cross-links [11]. Non-enzymatic cross-linking by AGEs decrease collagen’s susceptibility to degradation by matrix metalloproteinases, causing the build-up of collagen and subsequent stiffening of the usually pliant skeletal muscle ECM [12].

The endomysium ECM surrounding muscle fibers plays an important role in regulation of satellite cells, a muscle stem cell source, and in regulation of muscle health [13,14,15,16,17]. Within this endomysium are two laminae, the basal lamina and the reticular lamina. The basal lamina is ECM that contacts muscle fibers and is composed primarily of laminin, fibronectin, collagen type IV, and VI. The reticular lamina is ECM that separates conjoined basal laminae and is mainly composed of type I collagen that runs parallel to the muscle fibers, coalescing at the ends of the muscle into the tendons [12]. Skeletal muscle ECM plays a critical role in muscle function, and as such age-dependent changes in the ECM have considerable implications for muscle performance [18]. Furthermore, the basal and reticular laminae are important regulators of muscle regeneration by providing the appropriate spatiotemporal cues to satellite cells [13,19,20,21]. Increased fibrosis and stiffness of the aged laminae dysregulates this healing process and could hinder the ability of ECM to support myogenesis [14,15].

In young muscle, satellite cells are a resident stem cell population and endow muscle with a remarkable ability to regenerate, but there is little information about the impact of aged ECM on satellite cells and muscle regeneration [16,22,23,24]. Furthermore, less is known about whether AGE ECM cross-links could play an important role in muscle regeneration in older individuals with muscle injuries. The ECM is a critical mediator for satellite cell guidance cues and regulates the inflammatory response to determine whether fibrosis or regeneration occurs following injury. More, the regeneration of muscle injuries is dependent on the size of the injury itself, where larger injuries are more difficult to heal and often result in fibrosis [25]. Decellularized muscle matrix (DMM) is ideal for studying ECM-specific effects in muscle and as a regenerative tool to repair both small and large skeletal muscle injuries because its muscle-specific composition and anisotropy recapitulate the basal lamina environment. Decellularization is a method to procure an ECM scaffold rich in components specific to the tissue to which it is applied [26]. Skeletal muscle can be decellularized through several salt, enzymatic, and detergent soaks to lyse the cells, freeing them from the surrounding matrix, and washing them out of the tissue [25,26,27,28,29,30,31,32]. The white, ghost-like scaffolds that result encourage myogenesis due to muscle-specific moieties and lack of immunogenic material, making these scaffolds an ideal source to study age-dependent effects on muscle ECM [25,26,27,28,29,30,31,32]. Furthermore, DMM can be processed into versatile hydrogels useful for tissue engineering and other cell culture strategies because of its biocompatibility, body temperature-dependent polymerization, and potential as a cell delivery vehicle. The ability of DMM as a reviver of the myogenic process is dependent on the host’s age and immune capacity [33,34,35]. However, no studies have investigated whether deriving DMM from older individuals changed its myogenic characteristics, an important area of research that could underpin age-dependent ECM effects on muscle. We hypothesized that AGEs play a role in age-related collagen cross-linking and that those cross-links are retained in DMM sourced from old muscle. The aim of this study is to characterize age-dependent changes in AGE cross-linking in muscle and whether these changes persist in DMM.

## 2. Results

### 2.1. Histological Assessment of Whole Muscle

**Figure 1:**
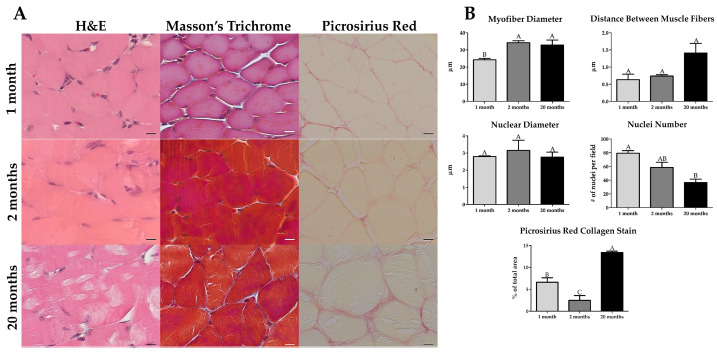
The gastrocnemius shows histomorphometric differences in old age. (**A**) Hematoxylin and Eosin (H&E) staining (n = 3) revealed a reduction in nuclei and the presence of lipofuscin-like or tubular aggregates inside the muscle fibers with old age. Masson’s Trichrome (n = 3) had no apparent differences with age. Picrosirius Red staining (n = 4) displayed a pronounced increase in collagen staining in old age. (**B**) When quantified histomorphometrically, the gastrocnemius had a smaller myofiber diameter in immature muscle, an increase in the distance between muscle fibers and a decrease in the number of nuclei in old age, and a bimodal amount of collagen staining where immature and old have higher levels of collagen than young adult, albeit older also has higher amounts than immature muscle. Scale bar = 10 µm. Groups that do not share a letter (e.g., **A**, **B**, or **C**) are statistically different according to a one-way ANOVA followed by a two-tailed Tukey’s correction (*p* < 0.05, n = 3 for H&E and Masson’s, n = 4 for Picrosirius Red).

We characterized the muscle before decellularization to determine if an aging phenotype was present. The myofiber diameter was noticeably smaller in 1-month samples compared to 2-month and 20-month. Nuclei were counted in all samples, and we determined there to be a reduction in the number of total nuclei with age. We also observed a lipofuscin-like deposition or tubular aggregates inside the myofibers at 20 months of age (Figure 1A) [36,37]. However, further histological investigations with lipid-detecting stains are needed to positively verify the presence of lipofuscin. In Masson’s trichrome, we did not observe any qualitative morphological differences. The variation in color shade and intensity in the Masson’s Trichrome is a technical staining artifact. Picrosirius red stains demonstrated a significant increase in collagen thickening and staining intensity at 20 months (Figure 1A).

With histomorphometry, we confirmed an increase in muscle fiber diameter from 1-month to 2 months of age, and we observed an increase, albeit not significant, in the distance between the muscle fibers at 20 months of age, and there was a reduction in the number of nuclei from 1-month to 20 months of age (Figure 1B and Supplementary Table S1). The area of Picrosirius red collagen stain was significantly elevated at 20 months compared to 1 and 2 months, also 1-month was significantly higher than 2 months (Figure 1B and Supplementary Table S1). Whole muscle sections, stained using Hematoxylin and Eosin (H&E) and Picrosirius red (Supplementary Figure S1A,B), showed an overall increase in muscle size between 1-month and 2-month, and 1-month and 20-month samples. Picrosirius red staining also showed increased collagen staining between 2- and 20-month muscles.

### 2.2. Age-Associated Effects in Whole Muscle Proteins

**Figure 2:**
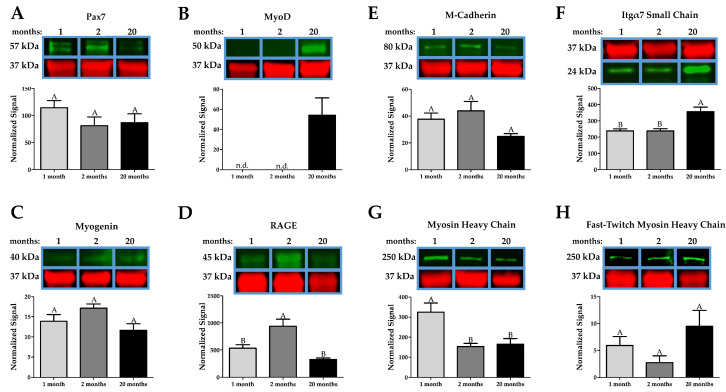
Western blotting differences in muscle-specific factors with age. (**A**,**B**) Pax7 does not change with age, suggesting that the satellite pool is unchanged at 20 months, while MyoD remains undetected (n.d. indicates not detected) until 20 months, indicating homeostatic compensation of the muscle. (**C**) Myogenin is marginally reduced at 20 months. (**D**) Curiously, RAGE (receptor for advanced glycation end products) decreased from 2 to 20 months. (**E**) M-cadherin is decreased in old age. (**F**) Integrin α7 is increased at 20 months, which might be a compensatory response by the muscle. (**G**) Myosin Heavy Chain is unchanged in old age compared to 2 months, indicating that muscle aging does not affect contractile units at 20 months. (**H**) There is no difference in the Fast-Twitch Myosin Heavy Chain, further indicating that 20 months is not old enough to see sarcopenic effects. The red bands are GAPDH. Groups that do not share a letter (e.g., **A** or **B**) are statistically different according to a one-way ANOVA followed by a two-tailed Tukey’s correction (*p* < 0.05, n = 6).

We examined the gastrocnemius muscles at different ages using Western blotting to determine if there was an age-dependent effect on muscle-specific proteins. Pax7 levels, a marker of satellite cells, were unchanged with age, indicating that the satellite pool was not affected (Figure 2A). MyoD was not detected in 1- and 2-month muscle but was upregulated at 20 months (Figure 2B). Myogenin was not different between the age groups (Figure 2C). Interestingly, RAGE was highest at 2 months and significantly lower at 1 and 20 months, indicating that RAGE may not play a significant role in our system (Figure 2D).

M-cadherin was reduced, albeit not significantly, at 20 months compared to 1 and 2 months (Figure 2E), while integrin α7 was upregulated at 20 months compared to 1 and 2 months (Figure 2F). Interestingly, myosin heavy chain was lower at 2 months and 20 months of age compared to 1-month, but there was no difference in myosin heavy chain staining between 2 and 20-month muscle lysates (Figure 2G). Fast-twitch, though not significantly different, had an elevated level at 20 months compared to 1 and 2 months (Figure 2H).

### 2.3. Histological Assessment of Decellularized Muscle

**Figure 3:**
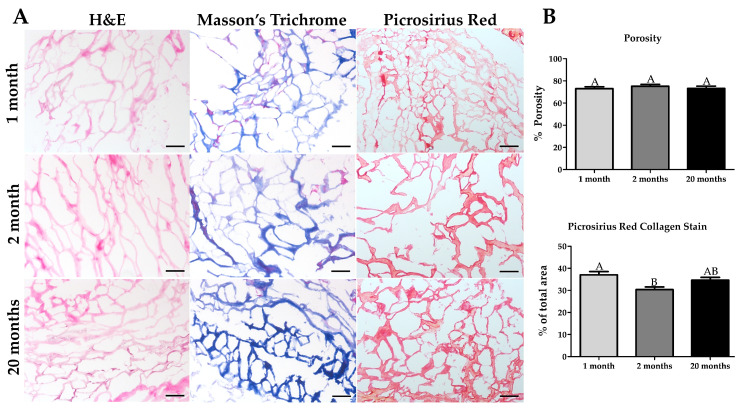
Decellularized muscle matrix (DMM) had no differences in collagen staining with old age. (**A**) Hematoxylin and Eosin (H&E) staining confirmed the absence of cell nuclei at all ages, indicating successful decellularization. Masson’s trichrome did not show age differences, while Picrosirius Red staining shows a thickening of the collagen with age in DMM. (**B**) There is no difference in porosity with age as measured with H&E staining. The amount of collagen area stained with Pricoririus Red staining is more significant in immature muscle compared to young adult. Although the mean area in old age is higher than in young adults, it is not statistically higher nor different from the immature muscle. Scale bar = 100 µm. Groups that do not share a letter (e.g., **A** or **B**) are statistically different according to a one-way ANOVA followed by a two-tailed Tukey’s correction (*p* < 0.05, n = 6).

We assessed DMM with histology to observe age-dependent changes to porosity and collagen content. Gastrocnemius muscles were decellularized and stained using hematoxylin and eosin. No nuclei were observed, indicating successful decellularization (Figure 3A).

To confirm decellularization, we measured levels of dsDNA in the pregel of the processed DMM gel and showed there was significantly less dsDNA in all age groups when compared to whole muscle (Supplementary Figure S2A). There was also no noticeable difference in Masson’s trichrome amongst the three age groups; however, Picrosirius red staining demonstrated increased collagen thickening in the 20-month group (Figure 3A) compared to 1, and 2-month decellularized muscle. Surprisingly, when we quantified Picrosirius red staining, we observed the highest levels of collagen staining in 1-month ECM. In contrast, sexually mature 2-month decellularized muscle staining was lower. While 20-month ECM stains were thicker and at higher levels, there was no statistical difference between 20 and 2 -months. ECM porosity was unchanged with age.

### 2.4. Ultrastructural Properties of Whole Muscle, DMM, and DMM Gels at Different Ages

**Figure 4:**
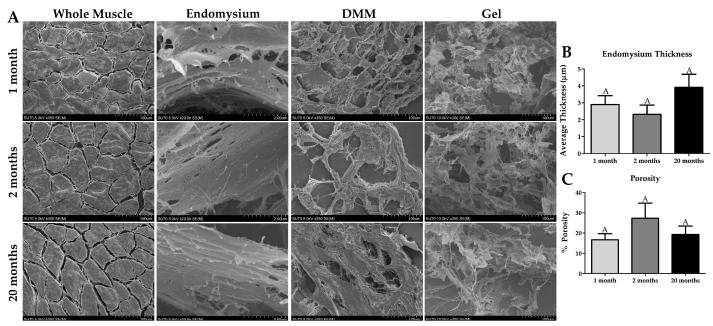
Scanning Electron Imaging shows distinctions in ECM structure with age in native muscle, decellularized muscle matrix (DMM), and processed DMM gels. (**A**) The muscle fibers are surrounded by endomysium in the whole muscle images, and the space between the muscle fibers appear to increase with age, however not significantly. DMM (n = 4 for 20-month DMM) and processed DMM gels, there is an obvious sclerotic structure indicating the presence of polymerized ECM such as collagen. (**B**) Endomysium thickness trends upwards with age but is not significantly different. (**C**) The porosity of DMM is not affected by age. Groups that do not share a letter (e.g., **A**) are statistically different according to a one-way ANOVA followed by a two-tailed Tukey’s correction (*p* < 0.05, n = 6 except the 20-month-old muscle has n = 4).

Cryosections were processed for scanning electron microscopy imaging to assess age-dependent changes in the ultrastructure of the ECM, and whether these changes persist in DMM. Muscle fibers in all whole muscle samples appeared normal with a polygonal morphology. We observed distinct areas of ECM between the muscle fibers as well, demonstrating successful imaging of the endomysium. Spacing between muscle fibers appeared to increase, however not significantly, while the endomysium remained unchanged between ages (Figure 4A,B).

In the processed DMM gels, we observed no differences in ultrastructure (Figure 4A). However, the gelation rate significantly decreased with increasing age, indicating that though collagen structure is similar differences in other age-dependent parameters such as collagen cross-linking may be at play (Supplementary Figure S2B). In addition, we measured porosity in DMM and did not observe any age-dependent differences (Figure 4C).

### 2.5. Age-Dependent Effects in Collagen Are Preserved in Processed Muscle

**Figure 5:**
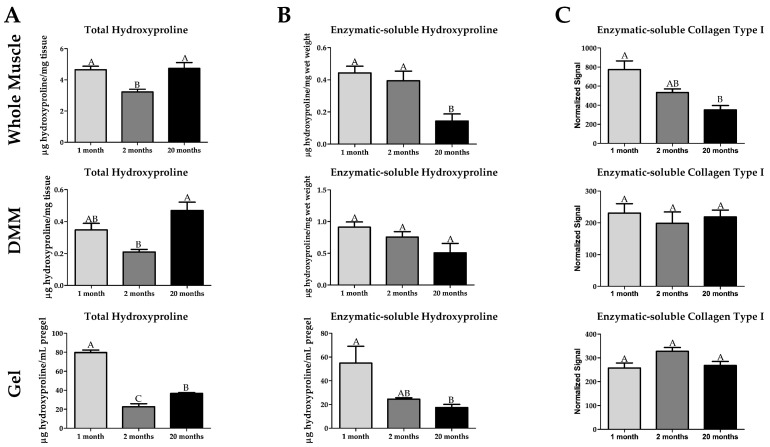
Total collagen is elevated in old age while soluble collagen is reduced as seen by hydroxyproline assays and SDS-PAGE. (**A**) Total hydroxyproline is increased at 1 month and 20 months compared to 2 months in whole muscle (n = 5 for 1 and 20 months, n = 6 for 2 months), decellularized muscle matrix (DMM) (n = 5 for 1-month, n = 6 for 2 and 20 months), and processed DMM gels (n = 3 for all groups), except 1 month is not higher than 2-month levels in DMM. Furthermore, total hydroxyproline levels are higher at 1 month in processed DMM gels compared to 20 months. (**B**) The amount of hydroxyproline soluble to the enzyme proteinase K is reduced in 20 months in whole muscle compared to 1 and 2 months and to 1 month in DMM (n = 5 for all groups) and processed DMM gels (n = 3 for all groups), indicating increased levels of cross-linking. (**C**) There is a decreased amount of proteinase K-soluble Collagen Type I at 20 months as determined by SDS-PAGE immunostaining compared to 1 month in whole muscle, indicating an increase in cross-linking. In DMM (n = 5 for all groups) and DMM processed gels (n = 3 for all groups), there is no difference in soluble Collagen Type I due to age, which could be due to the processing of the collagen denaturing the structural epitopes that the collagen antibody recognizes.Groups that do not share a letter (e.g., **A**, **B**, or **C**) are statistically different according to a one-way ANOVA followed by a two-tailed Tukey’s correction (*p* < 0.05, n value is detailed in each section).

To confirm the measured increase in Picrosirius red collagen staining with age and explore the age-dependent changes in collagen cross-linking, we used hydroxyproline-based assays. Hydroxyproline (the hydroxylated amino acid proline) is widely used as an indicator for the amount of collagen in tissue [38]. We measured increased total hydroxyproline amount in 20-month samples compared to 2-month in the whole muscle, DMM, and the processed DMM gels (Figure 5A). There was a higher amount of hydroxyproline in 1-month whole muscle and processed DMM gels (Figure 5A). To assay cross-linking, we digested our samples in proteinase K. Sample homogenates consisted of dissolved supernatant and a small pellet. We observed that the size of the pellet increased with sample age. Next, we quantified the amount of solubilized hydroxyproline in the supernatant. We detected reduced enzymatic-soluble hydroxyproline at 20 months in the whole muscles. A similar decrease was observed in DMM and the processed DMM gels, albeit only significant to 1-month samples in DMM gel (Figure 5B). To confirm our data, we processed the samples for SDS-PAGE and immuno-stained with a collagen type 1 antibody (which binds to the collagen molecule) and showed a significant decrease in the amount of collagen type 1 at 20 months compared to 1 month in the whole-muscle samples (Figure 5C). In contrast, collagen I in DMM and DMM gels was unchanged (Figure 5C). However, when we assayed the pregel solution, we observed a decrease in procollagen in the 20-month pre-gels compared to 2 months (Supplementary Figure S2C).

### 2.6. Increased Stiffness in DMM from Old Muscle

**Figure 6:**
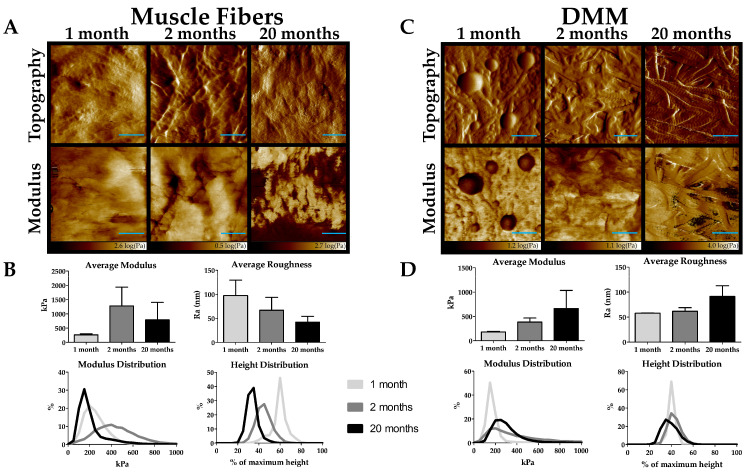
Atomic Force Microscopy imaging shows an increase in stiffness with age in decellularized muscle matrix (DMM). (**A**) The topography and modulus maps of muscle fibers show no noticeable difference with age. (**B**) The modulus of immature muscle fibers is reduced compared to 2 and 20 months. The average roughness of muscle fibers decreased with age, and the peak of the height distribution decreases with age. (**C**) The topography of DMM shows an increase in collagen structure with age. The topography of modulus is increased in old age. (**D**) The average modulus increased with age in DMM, and the peak of the modulus distribution is shifted to a higher value in old age. The average roughness is increased in old age compared to immature and young adult DMM. The peak of the height distribution is shifted slightly lower in old age. (n = 3 for all data in this figure). Scale bar = 500 nm.

We used atomic force microscopy imaging to test for age-dependent changes to muscle and DMM topography and stiffness. Cross-sectional muscle fiber topography was unchanged with age (Figure 6A). However, average fiber modulus was lower in 1-month muscle, and we observed a leftward shift in the modulus frequency distribution in 1-month muscle (Figure 6B). In addition, average roughness appeared to decline with age, and we observed an age-dependent decrease in percent maximum height (Figure 6B). In DMM, we demonstrated increased collagen structure with increasing age and an increased modulus with increasing age (Figure 6C). When we measured the average modulus, we detected an age-dependent increase in stiffness which was also observed in the modulus frequency distribution plots, where 20-month DMM was shifted toward higher moduli values compared to 1- and 2-month DMM (Figure 6D). In contrast to muscle fibers, average roughness increased in 20-month DMM compared to 1- and 2-month DMM. Height distribution was similar for 1- and 2-month DMM, while it appeared shifted to a lower maximum height for 20-month DMM (Figure 6D).

### 2.7. Advanced Glycation End Products Are Retained in DMM

**Figure 7:**
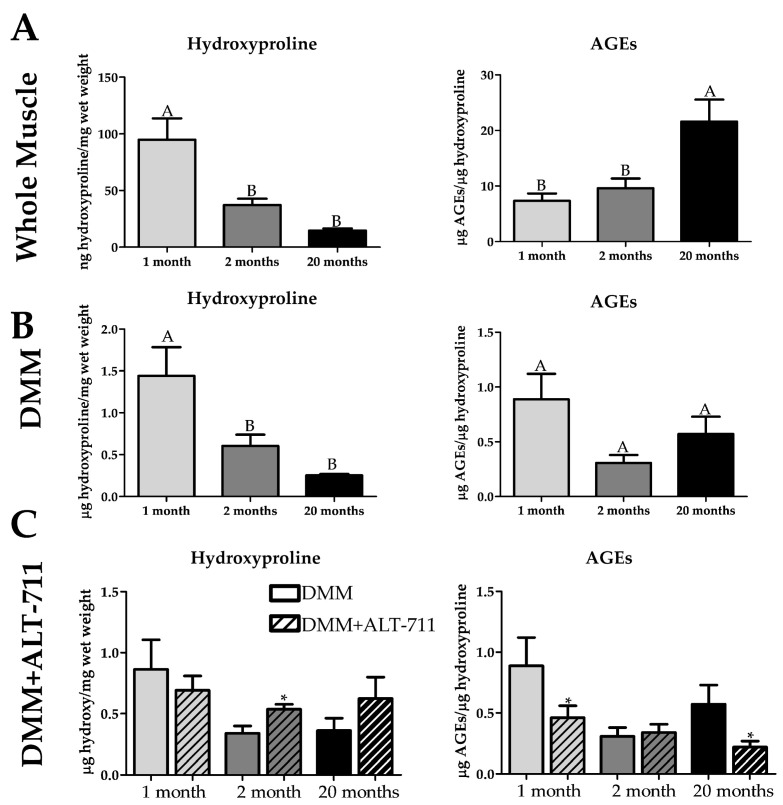
Advanced glycation end products (AGEs) are increased on old age in whole muscle but not in decellularized muscle matrix (DMM). However, the AGE cross-link breaker ALT-711 reduces the amount of AGEs in aged DMM. (**A**) The amount of detergent and mechanically soluble hydroxyproline is decreased in 2 and 20 months in whole muscle, indicating more cross-linking. AGEs are increased in old whole muscle relative to hydroxyproline (n = 6 in all groups). (**B**) The amount of detergent and mechanically soluble hydroxyproline is decreased in 2 and 20 months in DMM, indicating more cross-linking. However, there is no significant difference in AGEs relative to hydroxyproline with age in DMM (n = 5 for 1- and 2-month groups, n = 6 for 20 months). (**C**) After treatment with ALT-711, the amount of detergent and mechanically soluble hydroxyproline was increased in 2-month samples compared to untreated age-matched DMM, and there was a reduction in the number of AGEs per hydroxyproline in 1-month and 20-month DMM treated with ALT-711 compared to untreated age-matched DMM, indicating that AGE reduction was effective at all ages. Groups that do not share a letter (e.g., **A** or **B**) are statistically different according to a one-way ANOVA followed by a two-tailed Tukey’s correction (*p* < 0.05). * = statistically different to the age-matched control group (DMM without ALT-711) according to a two-tailed unpaired *t*-test (*p* < 0.05, n value detailed in each section).

To test whether the increased cross-linking we observed in whole muscle is due to glycation cross-links (AGEs), we used the AGE ELISA kit. We quantified the amount of mechanically soluble hydroxyproline, and AGEs were normalized to hydroxyproline in our samples. Mechanical solubilization is not as effective at extracting hydroxyproline using proteinase K due to the cross-links. This is supported by our data where we observed a decrease in the amount of mechanically soluble hydroxyproline in 2-month and 20-month whole muscle and DMM compared to the 1-month (Figure 7A). We demonstrated a significant increase in AGEs in 20-month whole muscle compared to 1- and 2-month muscle (Figure 7A). In contrast, we did not observe an age-dependent increase in AGEs in DMM (Figure 7B). No differences in total AGEs were observed in whole muscle and DMM, while there was a decrease in AGEs relative to wet weight in 2 and 20-month whole muscle and DMM samples compared to 1-month (Supplementary Figure S3A,C). A decrease in AGEs per protein was measured in whole muscle between 1-month and 20 months, while there was no difference in DMM (Supplementary Figure S3E). In addition, the wet weight increased with increasing age in whole muscle, while wet weight was reduced in 1-month compared to 2 and 20 months in DMM (Supplementary Figure S3B). The same amount of protein was extracted from each age in whole muscle, while more protein was extracted in 1-month DMM compared to the other groups (Supplementary Figure S3D). To further determine whether AGEs were responsible for reducing hydroxyproline with age, we treated DMM with an AGE cross-link breaker (ALT-711). ALT-711 incubation increased the amount of soluble hydroxyproline in 2-month and 20-month DMM, albeit only significant in the 2-month samples (Figure 7C). We also observed a significant decrease in the number of AGEs in the 1-month and 20-month group compared to the untreated age-matched DMM (Figure 7C). Lastly, we treated human DMM with ALT-711 and determined that ALT-711 reduced AGEs (relative to protein and hydroxyproline levels) in a clinically relevant human-derived DMM (Supplementary Figure S4A,B).

## 3. Discussion

The overall goal of this study was to determine if collagen and AGE accumulation in skeletal muscle was specifically found in muscle ECM and retained in DMM. We captured muscle characteristics during various stages of maturation and aging using 1-month, 2-month, and 20-month gastrocnemius muscles from *C57BL/6J* mice. Characterization of these muscle specimens was an important step to identify whether AGE accumulation was detectable in older whole muscle and if it was specifically increased in relation to muscle ECM. Additionally, we chose the gastrocnemius as it has a heterogeneous distribution of fiber types to capture aspects of slow and fast-twitch muscle aging [39,40]. Furthermore, the gastrocnemius is among the hind limb muscles reported to be more susceptible to the aging process in rats [41], and was shown to be affected in 70-week-old mouse models of McArdle disease [42]. Next, we examined how muscle ECM was affected by aging. To do so, we decellularized the muscles, confirmed a successful decellularization, and characterized these matrices. In support of our hypothesis, we determined that AGEs accumulated in both old whole muscle and decellularized muscle ECM, indicating that AGE cross-links were conserved following decellularization. This was an important finding because it demonstrated that AGE cross-links in muscle ECM were detected and could have further implications in aged satellite cell and muscle fiber biology.

Investigation of 1-month, 2-month, and 20-month muscle captured various stages of collagen aging. During muscle maturation, collagens are not fully cross-linked, allowing the ECM to have a soft and spacious environment [43,44,45]. However, at sexual maturity (~ 2 months in murine models), stable lysyl oxidase (LOX) cross-links form and persist throughout adult life. As we age, collagen cross-links slowly accumulate, but remain at low levels relative to tissue weight. At about 20 years in humans AGEs begin to accumulate at a constant rate, dictated by the specific collagen turn-over time of each tissue type, and this process continues into old age [46]. Furthermore, starting at about 70 years of age, AGE accumulation on muscle collagen is correlated to loss of muscle function [47]. For this reason, we selected 20-month muscle as our aging model because unlike extreme old age (geriatric) only some of the aging features are present, which is more representative of an average older population [48,49,50]. In addition, it was previously reported that increased levels of AGE cross-links in the body is an early indicator of later pathological aging events [51].

Furthermore, 20-month-old *C57BL/6J* mice are within the published accepted range for old age (18–24 months) with the maximum of 24 months based on 85% survivorship [52,53]. Selecting *C57BL/6J* mice older than 24 months is not advisable because of the increased prevalence of age-specific disease that could lead to confounding results [52,53]. 18–22-month-old *C57BL/6J* mice have also been published as “old” age groups in multiple tissue types, including the lung, gut, spleen, skin, ovaries, eyes, brain, nails, tendon, meniscus, cartilage, bone, and skeletal muscle [42,54,55,56,57,58,59,60,61,62]. Additionally, senescence-like changes, a marker of old age, are observed between 18 and 24 months in *C57BL/6J* mice [54,63,64,65].

Specific morphometric changes are expected in old age with skeletal muscles that reflect a reduction in cellularity with an increase in the ECM component [66]. We did not observe a decrease in the muscle fiber diameter with age, but there was a decrease in the number of nuclei, indicating that at 20 months an aging phenotype was not as pronounced as has been tested in other studies that use older mice [67]. The apparent increase in the amount of collagen at 20 months indicates changes in the ECM that precede changes in the muscle fibers themselves, even at this earlier stage of aging. In the immature 1-month group, we showed increased collagen levels that coincided with a decrease in muscle fiber diameter. Reduced dimensions of the muscle fibers could explain the increased amount of collagen relative to total area. However, it is also possible that the collagen occupied a larger volume due to less mature collagen cross-links [66,68]. This was confirmed quantitatively by measuring the amount of total hydroxyproline relative to weight at each age group.

Over time, there is an age-dependent decline in satellite cells that is associated with dysregulated differentiation and cell to cell fusion in the generation of new muscle fibers [69]. These age-dependent declines in the satellite cell pool are mirrored in the level of Pax7 detected in the tissue. Surprisingly, we did not measure a reduction in Pax7, suggesting that the number of satellite cells has not yet reached a sarcopenic decline at 20 months. However, another satellite and progenitor cell marker, m-cadherin, was reduced at 20 months of age. M-cadherin plays a role in maintaining the satellite cell niche in uninjured muscle and its decline could indicate that satellite cells may be at the beginning of sarcopenic decrease [70,71,72,73]. The transcription factors MyoD and myogenin are known to be upregulated in old age in a process known as homeostatic decompensation in a final attempt at recovering the muscle late in life [74,75,76]. Normally the myogenic transcription factors are at a low basal level in adult muscle, but when injury occurs, they are upregulated again and are responsible for patterning the myogenic process. Furthermore, in cases of denervation injury these transcription factors upregulate to form new muscle fibers in a recovery response to the atrophy and muscle fiber degradation typical to denervated muscle [74]. The aging environment in muscle is multifactorial but it is conjectured that the increase in MyoD and myogenin in aged muscle is in response to the retreat and fast-to-slow transition of the motor nerves [74]. Since MyoD was upregulated at 20 months and myogenin downregulated slightly, the gastrocnemius at 20 months of age may be in the beginning stages of homeostatic decompensation.

The receptor for advanced glycation end products (RAGE) is thought to play a role in skeletal muscle aging by mediating the proinflammatory effects of AGEs and other RAGE ligands [77]. While we expected RAGE levels to increase in older animals, RAGE levels were actually lower in 20-month lysates compared to 2-month lysates. In muscle, RAGE is primarily expressed in activated satellite cells and differentiated myoblasts. As myoblasts fuse with existing muscle fibers, RAGE expression decreases dramatically, indicating that its expression in muscle is related to the progenitor cell pool [78,79]. In addition, there is a decrease in the amount of RAGE measured in human satellite cells in old age, and this is partially due to an increase in truncation of RAGE that makes it not detectable via immunostaining [80]. Furthermore, RAGE levels in 2-month muscle could be vastly different compared to middle aged muscle. In order to fully determine why 20-month muscle was reduced compared to 2-month would require more study. 

Integrin α7 is a transmembrane protein responsible for transmitting force, is upregulated in differentiated myoblasts, and protects against sarcolemmal damage [81,82]. Integrin signaling has been implicated in muscle aging. Targeting β1-integrin signaling improved regeneration in dystrophic muscle and demonstrated that its deletion resulted in sarcopenic-like muscle [83]. Muscle’s β1-integrin counterpart, α7, has less known effects during aging. Recent evidence suggests no differences between levels of α7B in 3- and 22-month murine muscle [84]. In contrast, our data show an increase in total *α7* at 20 months, suggesting a compensatory mechanism to protect muscle from sarcolemmal damage. Whether differences in α7B also exist in our model was not investigated. Slow-twitch muscle fibers are known to increase relative to fast-twitch muscle fibers with age, especially in sarcopenia [85,86]. We did not observe an increase in the level of total myosin heavy chain nor did we observe a change in fast-twitch myosin heavy chain levels in 20-month muscle compared to 2-month samples, suggesting that the degree of age-related changes in muscle has not yet affected muscle fibers. This supports our use of 20-month aged mice in order to assess the accumulation of AGE-crosslinks independent of other mechanisms associated with frail, sarcopenic muscle phenotypes. In addition, changes in fiber composition were found to be subtle and specific to fast twitch fiber type in the early stages of aging [87]. Additionally, recent evidence reported that fast-twitch muscle from *C57BL/6J* mice displayed subtle changes in myofiber type composition at 24 months of age [36]. Furthermore, *C57BL/6J* mice are known to show age-dependent loss in muscular strength at an older relative age than humans, and as such may not be the best sarcopenic model [7,88].

Since we observed age-dependent changes in ECM, we anticipated that these could carry over to DMM. In support of our hypothesis, we observed a significant increase in collagen at 20 months in DMM, indicating that additional age-related cross-links attenuated normal collagen turnover. Skeletal muscle collagen is heavily cross-linked by glycation in aged muscles, stiffening the matrix and causing it to be resistant to enzymatic turn-over [10,19,20]. Furthermore, no considerable differences in enzymatic cross-linking have been observed with age [47,89]. To test whether cross-links were increased in old muscle, we digested our samples in proteinase K and measured the amount of soluble hydroxyproline. There was a reduction in enzymatic-soluble hydroxyproline in 20-month whole muscle, implying an increase in the amount of collagen cross-linking. Interestingly, this effect was lost after decellularization, indicating that decellularization destroyed collagen cross-linking or otherwise caused the collagen to be more susceptible to protease degradation. If aberrant collagen cross-linking is reduced by decellularization, this would be an advantage as DMM would not “remember” the age of its collagen. However, an alternative explanation is that DMM lacks intracellular proteins to inhibit the proteinase K, thus the collagen cross-linking is overtaken by proteolytic cleavage in this case. We further processed our DMM into a gel and observed that both 2 and 20 months had significantly less enzymatic-soluble hydroxyproline, which could be due to a concentrating of the heavily cross-linked, irreducible parts of the matrix. 

Collagen cross-linking significantly regulates the mechanical properties of tissues [90]. In old age, skeletal muscle stiffens and dysregulates the satellite cell niche, abrogating regeneration [19,20]. Whether this increase in stiffness is related to muscle fiber stiffness or the ECM is unclear. In addition, studies that investigate muscle stiffness in vivo rely on bulk material properties, which may miss minute mechanical features that cells would experience [20,91]. We used the AFM method to overcome these limitations of bulk mechanical testing and we observed that the increase in stiffness with age is specific to DMM, supporting our hypothesis that increased stiffness was ECM-related. Furthermore, since DMM retained a stiffer microenvironment, its use as a regenerative biomaterial may be compromised as muscle progenitor cells aberrantly proliferate on stiffer substrates. Furthermore, stiffness is known to alter mesenchymal differentiation, with stiffer substrates supporting osteoblastic pathways at the expense of adipogenic, chondrogenic, and myogenic pathways [92,93]. Interestingly, we observed a rougher microenvironment in old DMM, which conflicts with the known decrease in collagen tortuosity with age [19,94]. However, an overall dysregulation in the structure of the basal lamina does occur with old age [95]. These data confirm that pathological changes occurred in the old muscle ECM.

To determine whether the age-dependent changes we observed in DMM collagen were AGE specific cross-links we tested levels of AGEs in our samples and detected an increase in the number of AGEs in old whole muscle when normalized to hydroxyproline. We did not observe this increase in AGEs when normalizing to wet weight and protein. This observation suggested that AGE crosslinks might be concentrated in the ECM, leading us to choose collagen for normalization. A known difficulty in measuring AGE content is that most AGEs reside in the irreducible matrix, and as such a normalization factor (e.g., hydroxyproline) that accounts for the matrix components would account for this [96]. Interestingly, when we measured the amount of mechanically soluble hydroxyproline, it was reduced at both 2 months and 20 months of age. Since we did not detect a reduction at 2 months in the harsher proteinase K digestion, we suspected that collagen cross-linking at 20 months was greater than 2 months. We observed this same effect with hydroxyproline in the DMM; however, there was no increase in AGEs with age. In our homogenization schema, it is possible that in DMM, where the intracellular components are removed, the AGEs are in the pellet and thus are not measurable. To test this theory, we treated DMM with ALT-711, an AGE-specific cross-link breaker, and showed that there was no longer a reduction in hydroxyproline with age [97]. When we measured AGEs in the ALT-711 treated DMM, there was a reduction in the 1-month and 20-month samples compared to no treatment, showing that ALT-711 is effective in reducing elevated levels of AGEs. All of this together emphasizes that there were AGE cross-links in the DMM below our assay’s detection limits and that ALT-711 effectively reduced them in the aged DMM. Impressively, ALT-711 treatment was also a viable method at reducing AGEs in human-derived DMM, pointing to its clinical relevance. Reducing AGEs on aged DMM could restore the supple microenvironment of younger muscle ECM while eliminating the harmful effects of aberrant RAGE signaling. Furthermore, if successful this would open the gateway for using readily available old human muscle in decellularization applications. However, future studies are needed to characterize this treatment on aged DMM in promoting skeletal muscle regeneration.

## 4. Materials and Methods

Animal Model: 1-month, 2-month, and 20-month-old male *C57BL/6J* mice were selected to represent an immature, young adult, and old adult mouse. The gastrocnemius muscle was used for all experiments (one n is one muscle). Gastrocnemius muscles were large enough to perform multiple assays. All surgical procedures were performed under an IACUC-approved protocol at VCU (AD10000675, 1 March 2019). Briefly, the mice were euthanized with CO_2_ asphyxiation followed by cervical dislocation. The skin covering the limbs was bluntly dissected, and the gastrocnemius muscles excised, taking care to remove the overlying biceps femoris and underlying soleus and plantaris. The Achilles tendon was also removed before experimental use.

Decellularization: Gastrocnemius from 1-month, 2-month, and 20-month-old male C57Bl/6 mice were used as a model of skeletal muscle aging. The gastrocnemius was isolated and frozen at −80 °C until decellularization. On the day of decellularization, the muscle was thawed and cut into quarters, and each quarter of the muscle was put into its own well of a 24-well plate and decellularized at 4 °C on a plate shaker (Supplementary Figure S5). The quarters were treated with three 10 min DI water washed, 0.25% trypsin for 6 h, 0.1% Triton X-100 for 24 h, 0.2% sodium deoxycholate for 24 h, DNase for 1 h, and 0.1% peracetic acid for 24 hours. There were three 15 min DI water washing steps between all steps, and five 15 min 1×PBS washes after the peracetic acid step. All volumes were 1.5 mL, except the DNAse was 0.5 mL. To create a DMM gel, the DMM was lyophilized and cryomilled to create a DMM powder. This powder was digested with pepsin (2300 units/mg powder) in 0.01M HCl for 6 hours, pH and salt balanced with NaOH and 10xPBS, and gelled at 37 °C for 1 h. Decellularization was confirmed by running the pre-gel on the picogreen dsDNA assay [98]. The gelation rate was determined by measuring optical density at 550 nm in a 96-well plate every 2 min at 37 °C for 1 h (n = 12 for all groups). The results were then curve-fitted using Matlab to extract the gelation rate [99]. Matlab code is available upon reasonable request.

Histological Evaluation: Whole muscle was processed with formalin-fixed paraffin-embedded (FFPE) tissue processing. Briefly, the tissue was fixed in 10% neutral-buffered formalin and then processed through increasing gradients of ethanol, cleared with xylenes, and infiltrated with paraffin wax. The samples were then embedded in paraffin wax, and 7 μm sections were taken, floated on a warm water bath, and picked up onto charged glass slides, dried, stained, and cover slipped. Before staining, the sections were deparaffinized with xylenes and rehydrated with decreasing gradients of ethanol. DMM was processed with cryosectioning, where the tissue was embedded in Optimal Cutting Temperature (OCT) compound, frozen at −80 °C, sectioned in a microtome at 7 μm onto charged glass slides, and kept at −80 °C until staining. Before staining, the OCT was washed out with DI water. Hemotoxylin and Eosin (H&E) and Masson’s Trichrome (Weigert’s Iron Hematoxylin, Biebrich Scarlet-Acid Fuchsin, and Aniline Blue) were applied to assess the muscle (3 representative 10× images per sample) and DMM (3 representative 10× images per sample) morphometrically. Additionally, 40× pictures were taken to represent the whole muscle in Figure 1. Masson’s Trichrome and Picrosirius Red were used to qualify and quantify, respectfully, the collagen content of whole muscle and DMM. Two 50× images per sample were quantified in the whole muscle Picrosirius Red sections, and three 10× images for DMM. The regions were selected based on representation (whether the image reflected the natural variation in the muscle or not) and usability for data analysis (e.g., regions where the muscles were cut longitudinally were not included, as it is not possible to measure the diameter). The Zen Pro software (Carl Zeiss Meditec AG, Jena, Germany) was used to perform the histomorphometry. Minimum Feret diameter was found for the whole muscle by using the linear measurement tool on Zen Pro to measure the minimum diameter of the myofibers [54,100]. The distance between myofibers was found using the linear measurement tool on Zen Pro. The collagen area was measured with the polygon contour tool for whole muscle in the Picrosirius red section. For decellularized muscle, the white area from the Picrosirius red images was measured using automatic thresholding using Zen Pro, and this was subtracted from the total area to find the area of red stain. Porosity of H&E stained DMM was found by measuring the empty spaces in 3 random 10× images per sample (Zeiss AxioVision Microscope; Carl Zeiss Microscopy LLC, White Plains, NY, USA), and then the data was presented as a percentage of these spaces to the total area of the image, averaged per sample. The same process was applied to the Picrosirius Red samples, except the red areas (collagen-stained) were quantified and reported as a percentage of the total area of the image, averaged per sample.

Scanning Electron Microscopy: Scanning Electron Microscopy (SEM) was used on tissue that is cryosectioned at 30 μm, washed with DI water to remove the OCT, lyophilized, and sputter-coated with platinum. The sections were imaged with SEM (Hitachi SU-70 FE-SEM, Hitachi, Ltd., Tokyo, Japan) with 5.0kV at × 350 and × 20.0k to characterize the ultrastructure. Zen Pro software (Carl Zeiss Meditec AG) was used to quantify the thickness of the endomysium and the porosity on x350 images. The porosity was found by measuring the total open pore area and dividing that area by the total area using the Zen Pro linear measurement tool for endomysium thickness, and using the contour polygon area tool.

Collagen Cross-linking: Collagen cross-linking was determined by assessing collagen’s resistance to Proteinase K digestion. Briefly, the samples were homogenized in NP-40 lysis buffer with a 6.0 mm zirconium bead in the beadbug homogenizer, then incubated in 800 U/mL Proteinase K (P8107S, New England Biolabs, Ipswich, MA, USA) for 1 h at 37 °C. A protease inhibitor cocktail was added, the samples were centrifuged at 13,000 rpm (17,949 g) (Centrifuge 5427 R, Eppendorf, Hamburg, Germany) for 3 min, and the supernatants hydrolyzed for 24 h with 5N HCl at 120 °C before being run on the hydroxyproline assay (6017, Chondrex, Woodinville, WA, USA). The supernatants were also assessed with Western blotting, where the rabbit anti-Collagen I antibody (ab34710, Abcam, Cambridge, UK) was used, and the results were normalized to total protein (926-11016, LI-COR Biosciences, Lincoln, NE, USA). This Western blotting approach was also applied to the pregel of the processed DMM gel. Other samples were hydrolyzed without homogenization to determine the total hydroxyproline amount (DMM was normalized to dry weight before decellularization).

Atomic Force Microscopy: Atomic Force Microscopy was used on tissue that is cryosectioned at 7 μm. The OCT was removed with DI water, and the section was hydrated with 1×PBS for the experiment’s duration. The Dimension FastScan AFM from Bruker along with the SCANASYST-FLUID tip (20 nm radius) was used to gather topographical and modulus measurements from the sections under 1×PBS. Each tip was calibrated under 1×PBS using thermal tuning followed by ramping (5 curves, 200 nm ramp height, 0.20 V ramp set point) to determine the k value. Then, the samples were probed using peakforce quantitative nanomechanical mapping under fluid (1×PBS) with a 2 µm × 2 µm scan size at 400 samples/line for topographic images. The same image was taken again but at 128 samples/line to gather individual force curves (16,384 per image). The modulus was extracted from each force curve by applying the 2-point JKR model with taking into account positive adhesion forces (Poisson’s ratio was assumed to be 0.5). The incalculable force curves were removed using MatLab (code available upon reasonable request) and the data averaged, then the histograms of the modulus and height values were plotted using GraphPad Prism (GraphPad Prism 5.04, GraphPad Software, San Diego, CA, USA).

AGEs: Muscle samples were minced and homogenized with the Minute^TM^ Total Protein Extraction Kit for Muscles (using the Denaturing Buffer) and ran on an AGE ELISA (STA-817, Cell Biolabs, San Diego, CA, USA) to determine AGE levels. For DMM, homogenization was done with a 6.0 mm zirconium bead in a beadbug homogenizer (BeadBug™ Cat #: 31-212, Genesee Scientific, San Diego, CA, USA) at 4000 rpm for 60 seconds 20 times while keeping the tubes on ice for at least 5 min between runs, to pulverize the tough ECM, followed by centrifugation at 13,000 rpm (17,949 g) (Centrifuge 5427 R, Eppendorf, Hamburg, Germany) for 3 min. For human DMM, homogenization was done with NP-40 lysis buffer with a 2 mL tenbroeck glass-pot homogenizer followed by centrifugation at 13,000 rpm (17,949 g) (Centrifuge 5427 R, Eppendorf, Hamburg, Germany) for 3 min. The Pierce™ BCA Protein Assay Kit (23,225 and 23227, Thermo Fisher Scientific, Waltham, MA, USA) and hydroxyproline assays were used for normalization purposes. To reduce AGE cross-links, DMM was incubated with 10 mM of the AGE-cross-link breaker ALT-711 (Alagebrium Chloride A3166 TCI America, Portland, OR, USA) in 1xPBS for 5 days at 37 °C. The DMM was then washed 3 times with DI water for 5 min each, then frozen at −80 °C until analysis.

Western Blotting: 30 mg of gastrocnemius muscles were homogenized in NP-40 lysis buffer (BP-119, Boston BioProducts, Ashland, MA, USA) with a PI cocktail and 25 mM NaF with a 6.0 mm zirconium bead in a beadbug homogenizer (BeadBug™ Cat #: 31-212, Genesee Scientific, San Diego, CA, USA) at 4000 rpm for 60 s 5 times while keeping the tubes on ice for at least 5 min between runs. The homogenate was centrifuged 9703 rpm (10,000 g) (Centrifuge 5427 R, Eppendorf, Hamburg, Germany) for 10 min, and the supernatant was used for Western blotting. Briefly, the supernatant was run on the BCA assay. Equal amounts of protein were denatured with Laemmli buffer at 100 °C for 10 min, then electrophoresed on polyacrylamide gels, transferred to a PVDF low fluorescence membrane, blocked for 1 h at room temperature, and stained overnight with primary antibodies (mouse anti-PAX7 (ab55494 Abcam, Cambridge, UK), mouse anti-MYOD (MA1-41017, Thermo Fisher, Waltham, MA, USA), mouse anti-Myogenin (ab1835, Abcam, Cambridge, UK), mouse anti-Myosin Heavy Chain clone A4.1025 (05-716, Sigma Aldrich, St. Louis, MO, USA), mouse Anti-Myosin (Skeletal, Fast) clone MY-32 (M4276, Sigma-Aldrich, St. Louis, MO, USA) rabbit anti-M Cadherin (ab87374, Abcam, Cambridge, UK), rabbit anti-ITGA7 (PA5-37435, Thermo Fisher, Waltham, MA, USA), rabbit anti-RAGE (ab37647, Abcam, Cambridge, UK) or rabbit anti-GAPDH (14C10) (2118S Cell Signaling Technology, Danvers, MA, USA). The membranes were then incubated with secondary antibodies (926-68073, 926-32210, and 926-32211, LI-COR Biosciences, Lincoln, NE, USA) for 40 min at room temperature, then they were imaged on the LI-COR Odyssey and quantified. 1-month and 2-month samples were run on the same gel, and 20-month samples were run on a separate gel. Each blot was analyzed separately and results were normalized to GAPDH levels. The normalization factor for samples on one blot was determined by identifying the highest GAPDH signal and dividing each GAPDH signal intensity by the highest GAPDH signal, producing a value of 1 for the strongest signal and less than 1 relative to that stronger signal. Target protein signals were divided by the normalization factor to obtain the normalized signal. An advantage of this normalization method is that it is not affected by variations in housekeeping proteins as seen in old age [101].

Statistical Analysis: Data are represented as mean ± standard error of the mean. Statistical analysis is performed using a one-way analysis of variance (ANOVA) followed by a two-tailed Tukey’s correction to determine significance using α = 0.05. Groups not sharing letters are statistically significant. Outliers were removed using Grubb’s test (α = 0.05). All statistical analyses are performed using GraphPad Prism (GraphPad Prism 5.04, GraphPad Software, San Diego, CA, USA) and JMP Pro (JMP Pro 15, SAS Institute, Cary, NC, USA).

## 5. Conclusions

Skeletal muscle aging elevates the amount of intramuscular collagen, AGE cross-linking, and stiffness at a clinically relevant age that could be deleterious to regeneration. Our data shows that these aging alterations are preserved in DMM, providing an obstacle for the clinical translation of DMM since older muscle is available in greater frequency. Interestingly, these changes are also retained in a more processed DMM gel, indicating that age still affects DMM at every level of reduction. However, ALT-711 shows efficacy in reversing AGE cross-linking in DMM, providing a pathway for future biological studies at improving aged DMM.

## Supplementary Materials

The following are available online at https://www.mdpi.com/article/10.3390/ijms22168832/s1.
