# Supplementary material for: Advanced Glycation End Products Are Retained in Decellularized Muscle Matrix Derived from Aged Skeletal Muscle
Source: Int J Mol Sci. 2021 Aug 17;22(16):8832. doi: 10.3390/ijms22168832 (PMC8396213; doi:10.3390/ijms22168832)
Supplement: Supplementary file 1 [file ijms-22-08832-s001.zip › ijms-1271678-supplementary.pdf]

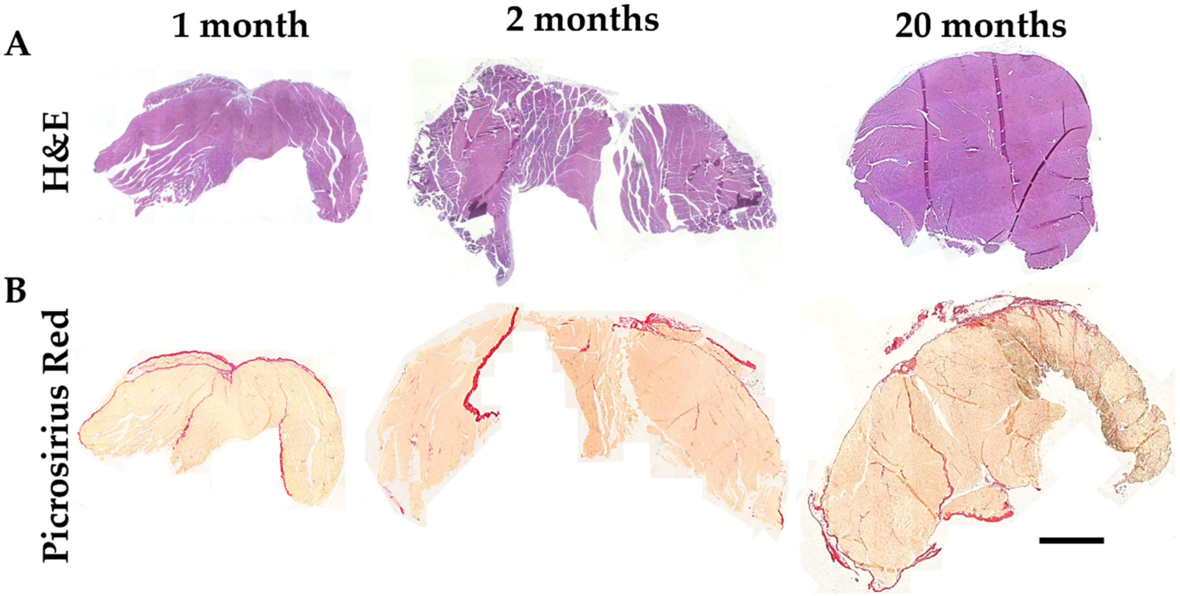

Supplementary Figure S1: Whole histological cross-sections of gastrocnemii of differing ages stained with H&E and Picrosirius Red. (**A**) H&E stained cross-sections show an increase in muscle size when comparing 2 and 20 months to 1 month. (**B**) Picrosirius red cross-sections. The scale bar is 1 mm and applies to all images.

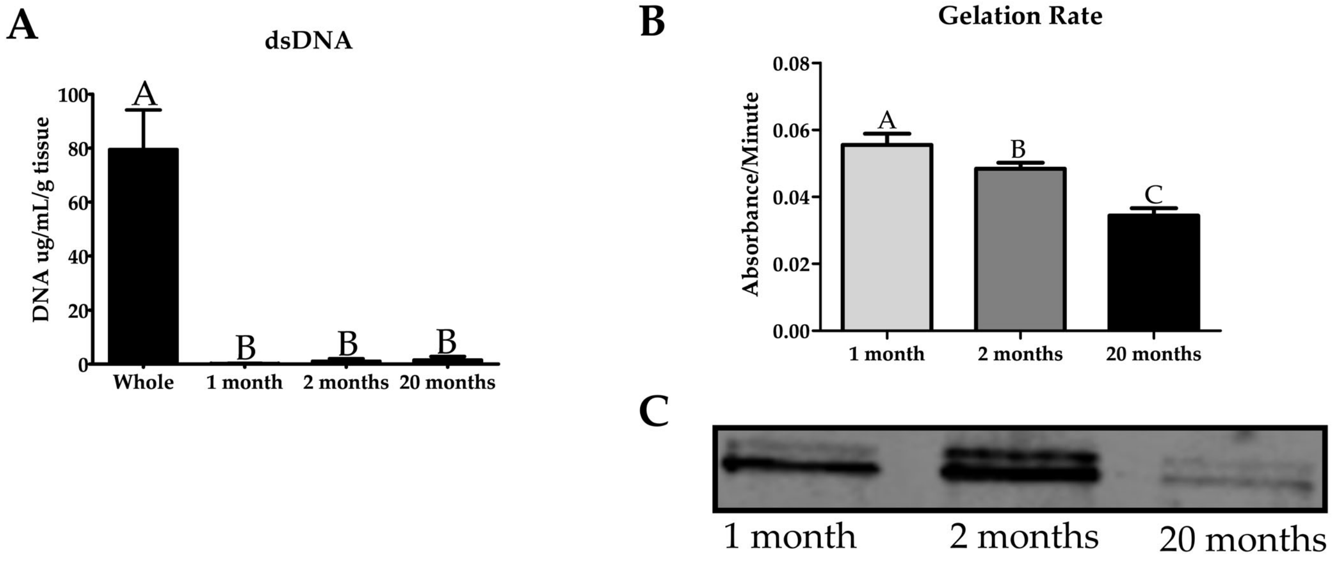

Supplementary Figure S2: Processed DMM gels are successfully decellularized and show age-dependent reductions in gelation rate and soluble procollagen. (**A**) dsDNA levels in 1-month, 2-month, and 20-month DMM pregels are significantly reduced compared to whole muscle. (**B**) Gelation rate significantly decreases with increasing age, indicating a reduction in collagen polymerization ability with age. (**C**) Procollagen was reduced in 20-month DMM pregels compared to 1 and 2-months.

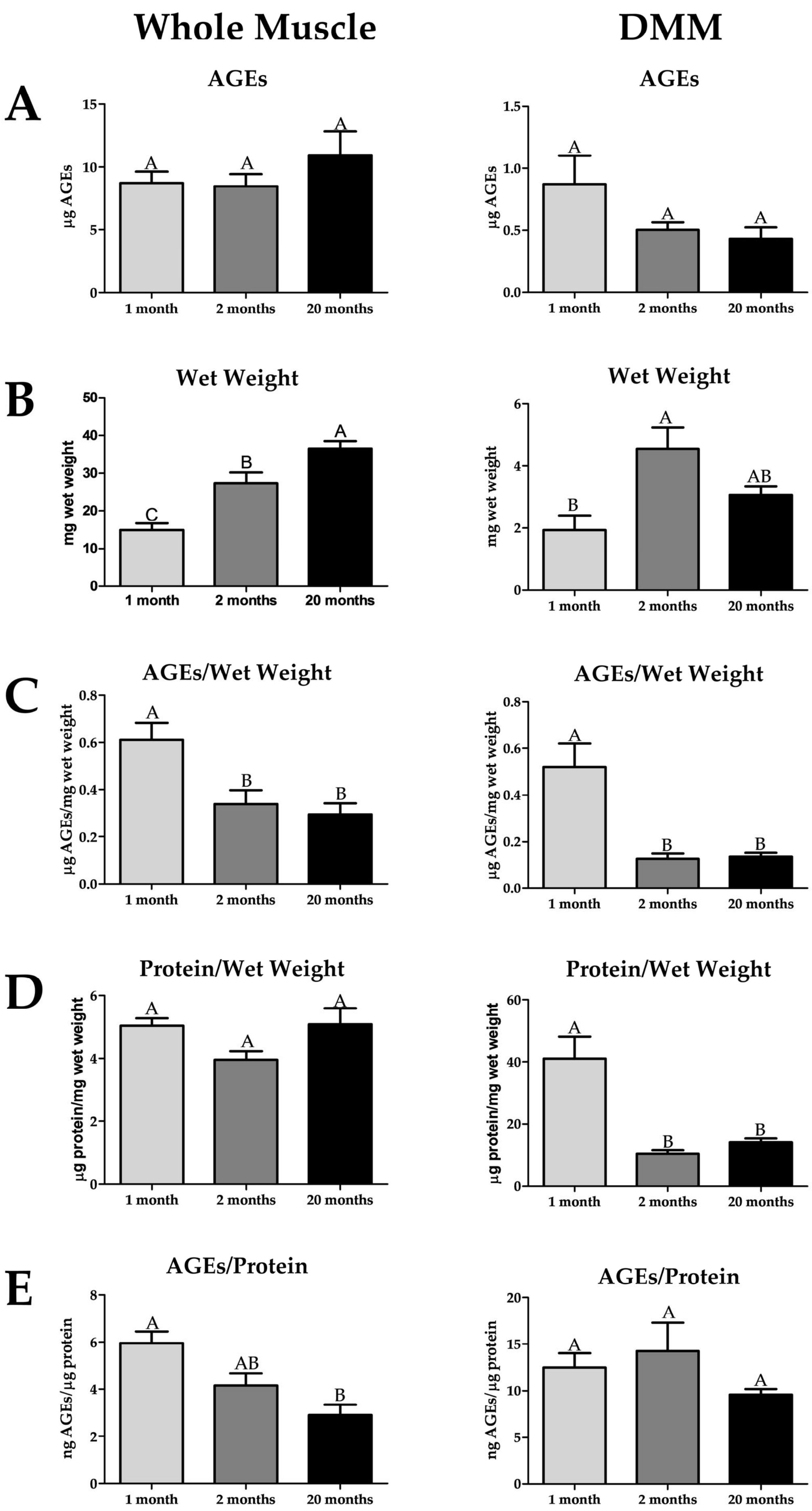
Whole Muscle
DMM
A
AGEs
µg AGEs
15
10
5
0
1.5
1.0
0.5
0.0
A
A
A
A
A
A
1 month
2 months
20 months
B
Wet Weight
mg wet weight
50
40
30
20
10
0
6
4
2
0
C
B
A
B
A
AB
C
AGEs/Wet Weight
µg AGEs/mg wet weight
0.8
0.6
0.4
0.2
0.0
A
B
B
A
B
B
D
Protein/Wet Weight
µg protein/mg wet weight
6
4
2
0
60
40
20
0
A
A
A
A
B
B
E
AGEs/Protein
ng AGEs/µg protein
8
6
4
2
0
20
15
10
5
0
A
AB
B
A
A
A

Supplementary Figure S3: Absolute AGE levels with differing normalization methods in whole muscle and DMM. (**A**) Absolute AGE levels (no normalization) did not change in whole muscle and DMM between different ages. (**B**) Wet weight of the samples increases with age in whole muscle, and increases from 1-month to 2-month in DMM. (**C**) AGEs per wet weight decreased from 1-month levels in the 2-month and 20-month groups for both whole muscle and DMM. (**D**) Protein extracted per weight was not different for whole muscle, but decreased in the 2-month and 20-month DMM compared to 1-month DMM. (**E**) AGEs per protein decreased in the 20-month whole muscles compared to 1-month, and there was no change for DMM. Groups that do not share a letter (e.g. A, B, or C) are statistically different according to a one-way ANOVA followed by a two-tailed Tukey's correction.

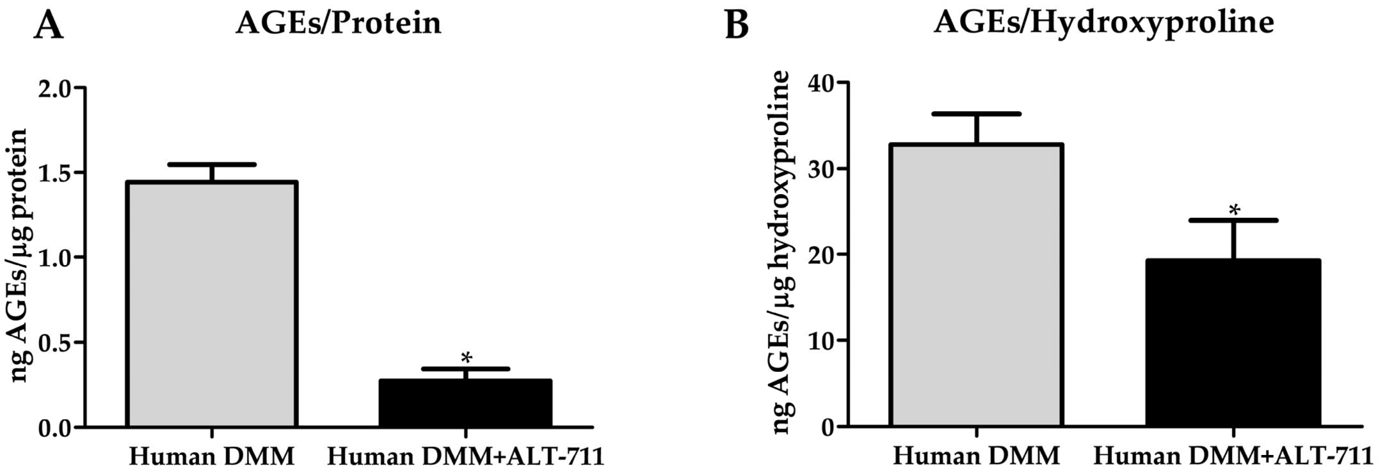

Supplementary Figure S4: ALT-711 treatment reduces AGEs in human DMM when normalized to protein and hydroxyproline levels. (**A**) AGEs per protein was reduced in human DMM is treated with ALT-711. (**B**) AGEs per hydroxyproline was reduced in human DMM treated with ALT-711. * = statistically different to the control group (DMM without ALT-711) according to a two-tailed unpaired t-test ($p < 0.05$).

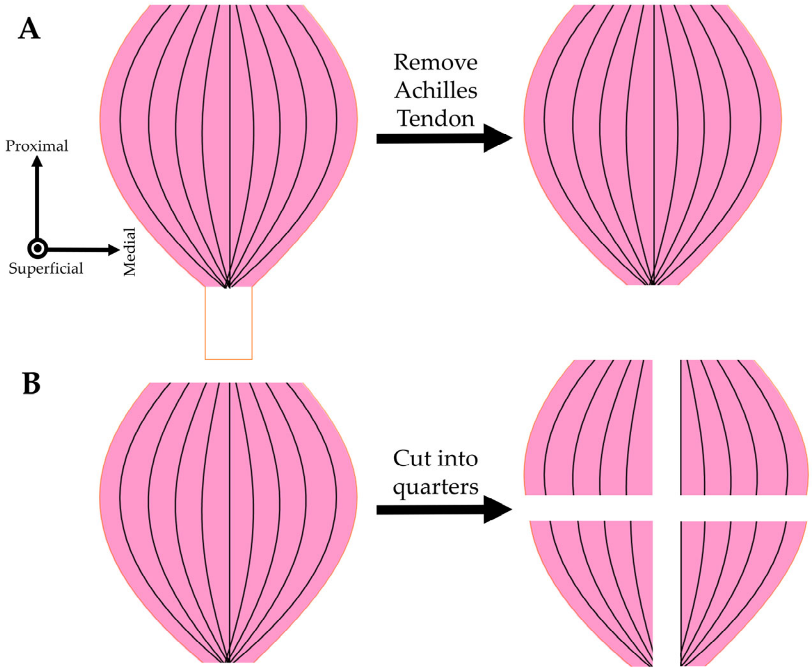

Supplementary Figure S5: Whole muscle preparation for decellularization. (**A**) The Achilles tendon was removed. (**B**) The gastrocnemius was cut into quarters.

Table S1: Number of myofibers, nuclei counted and measurement error for histomorphometry.

Group	# of myofibers (mean+/-SEM)	Myofiber diameter (mean+/-SEM)	# of nuclei (mean+/-SEM)
1 month	25+/-5.57	24.25+/-0.82 μm	79.33+/-3.84
2 months	11.33+/-0.33	34.27+/-1.12 μm	58.67+/-7.42
20 months	13.33+/-2.03	32.90+/-2.84 μm	36.67+/-4.91
